# Disinhibition-Like Behavior Correlates with Frontal Cortex Damage in an Animal Model of Chronic Alcohol Consumption and Thiamine Deficiency

**DOI:** 10.3390/biomedicines10020260

**Published:** 2022-01-25

**Authors:** Marta Moya, Leticia López-Valencia, Borja García-Bueno, Laura Orio

**Affiliations:** 1 Department of Psychobiology and Methods in Behavioral Sciences, Faculty of Psychology, Complutense University of Madrid (UCM), 28223 Madrid, Spain; martamoyamontes@ucm.es (M.M.); letlop01@ucm.es (L.L.-V.); 2 Red de Trastornos Adictivos (RTA), Instituto de Salud Carlos III (ISCIII), 28029 Madrid, Spain; 3 Departament of Pharmacology and Toxicology, Faculty of Medicine, Complutense University of Madrid (UCM), 28040 Madrid, Spain; bgbueno@med.ucm.es; 4 Instituto de Investigación Sanitaria Hospital 12 de Octubre (Imas12), Instituto Universitario de Investigación en Neuroquímica IUIN-UCM, Avda. Complutense s/n, 28040 Madrid, Spain; 5 Centro de Investigación Biomédica en Red de Salud Mental, Instituto de Salud Carlos III (ISCIII), 28029 Madrid, Spain

**Keywords:** chronic alcohol, thiamine deficiency, disinhibition, Wernicke’s encephalopathy, recognition memory, nitrosative stress, lipid peroxidation, apoptosis, cell damage, nutritional deficit

## Abstract

Wernicke–Korsakoff syndrome (WKS) is induced by thiamine deficiency (TD) and mainly related to alcohol consumption. Frontal cortex dysfunction has been associated with impulsivity and disinhibition in WKS patients. The pathophysiology involves oxidative stress, excitotoxicity and inflammatory responses leading to neuronal death, but the relative contributions of each factor (alcohol and TD, either isolated or in interaction) to these phenomena are still poorly understood. A rat model was used by forced consumption of 20% (*w*/*v*) alcohol for 9 months (CA), TD hit (TD diet + pyrithiamine 0.25 mg/kg, i.p. daily injections the last 12 days of experimentation (TDD)), and both combined treatments (CA+TDD). Motor and cognitive performance and cortical damage were examined. CA caused hyperlocomotion as a possible sensitization of ethanol-induced excitatory effects and recognition memory deficits. In addition, CA+TDD animals showed a disinhibited-like behavior which appeared to be dependent on TDD. Additionally, combined treatment led to more pronounced alterations in nitrosative stress, lipid peroxidation, apoptosis and cell damage markers. Correlations between injury signals and disinhibition suggest that CA+TDD disrupts behaviors dependent on the frontal cortex. Our study sheds light on the potential disease-specific mechanisms, reinforcing the need for neuroprotective therapeutic approaches along with preventive treatments for the nutritional deficiency in WKS.

## 1. Introduction

Alcohol is one of the most widely used psychoactive drugs worldwide, whose consumption originates major public health problems in our society including alcohol use disorder (AUD) (reviewed in [1]). The confluence of nutritional deficiency in AUD patients is of particular interest, specifically in the case of thiamine deficiency (TD), as this can result in Wernicke–Korsakoff syndrome (WKS). WKS is often characterized by ataxia, oculomotor disturbances, severe memory impairment, global confusion, executive dysfunction and apathy, mainly seen in chronic alcohol consumption cases. Ethanol affects thiamine bioavailability by multiple mechanisms such as reducing effective absorption, causing defective phosphorylation (thiamine diphosphate is the metabolically active form) and reducing thiamine storage in the liver because of alcoholic liver disease [2]. Approximately 75% of TD cases are undiagnosed in AUD patients (reviewed in [3]). Other causes inducing the pathology, independent of alcohol, include chronic malnutrition caused by prolonged fasting and hyperemesis gravidarum, bariatric and gastrointestinal surgeries, acute pancreatitis and hemodialysis [4,5].

Thiamine is central to the preservation of the myelin sheath, neurotransmitter synthesis and cellular energy reserves. Impaired oxidative metabolism in TD due to decreased activity of thiamine-dependent enzymes leads to a multifactorial cascade of events in the brain that includes focal decreases in energy status, decreased glucose utilization, lactic acidosis, blood–brain barrier disruption, astrocyte dysfunction, glutamate-mediated excitotoxicity, amyloid deposition, oxidative stress and inflammation [6].

WKS patients have obvious cognitive and motor dysfunction, requiring heavy dependence to complete daily life activities. The most apparent neuropsychological deficits are memory alterations. However, these alterations are not the only ones, and on many occasions, they are not the most limiting. The important alterations that they suffer in the field of affectivity and executive functions are much more devastating than memory problems. These patients frequently show affective flattening and lack the drive to start activities, which implies a loss of spontaneity and initiative [7,8]. Poor risk assessment and altered behavioral inhibition are also frequently encountered [9]. Disinhibition is a debilitating problem leading to impulsive and socially inappropriate behavior [10]. Thus, the set of neuropsychological characteristics indicated suggests a dysfunction of the frontal structures. There are several neuroimaging studies that indicate alterations in the frontal lobes of patients with WKS [11,12,13,14]. The marked apathy and lack of initiative observed in patients with WKS has also been found in many subjects with frontal injuries [15].

Likewise, evidence indicates that the frontal cortex is important in behavioral inhibition, including cognitive processes, social behavior and inhibition of motor responses. Damage to the frontal cortex lowers performance in executive control tasks, most likely by disrupting inhibition [10].

The frontal cortex is a structure vulnerable to damage from both alcohol and TD, as has been described in previous studies (reviewed in [16]) including some by our research group [17,18].

In uncomplicated AUD patients and comorbid AUD patients with WKS, the prefrontal cortex is more vulnerable to chronic ethanol exposure than other cortical regions [19], as there are decreases in neuron density (15–23%) [20], shrinkage of the frontal lobe volume [21] and decreased metabolic rates (reviewed in [16]). Similar neuropathological changes in the frontal cortex have been detected in non-alcoholic WKS patients [22].

Phenomena such as oxidative or nitrosative stress, lipid peroxidation, excitotoxicity and inflammation have been associated with cell damage and neurodegeneration induced by chronic alcohol and TD in this brain region (reviewed in [1,16]).

Thus, both ethanol toxicity and TD result in cortical damage and cognitive problems, but the relative contributions of each factor to these phenomena and the underlying mechanisms are still poorly understood.

Here, we examined the effects of chronic alcohol exposure (9 months), a TD diet and the combination of both in terms of the neurotoxic parameters (nitrosative stress, lipid peroxidation, apoptosis and cell damage) in the frontal cortex and behavioral tasks that are highly dependent on this brain region to further understand the independent effects of each treatment and how they interact.

## 2. Materials and Methods

### 2.1. Animals and Housing

Male Wistar rats (Envigo©, Barcelona, Spain) (*n* = 50) weighing 100–125 g at arrival (postnatal day (PD) 28, approximately) were used. Animals were housed in groups of 2–3 per cage and maintained at a constant room temperature (21 ± 1 °C) and humidity (60 ± 10%) in a reversed 12-h dark–light cycle (lights on at 8:00 p.m.). Standard food and tap water were available *ad libitum* during an acclimation period of 12 days prior to experimentation, and then the rats were randomly assigned to the experimental groups. Thus, the animals began the experiments when they were around PD 40 (adolescent period). The number of animals in the alcohol and TD groups was slightly higher than that of the control groups for the possible loss of experimental subjects.

All procedures followed ARRIVAL guidelines and adhered to the guidelines of the Animal Welfare Committee of the Complutense University of Madrid (reference: PROEX 312-19) in compliance with Spanish Royal Decree 53/2013 and following European Directive 2010/63/EU on the protection of animals used for research and other scientific purposes.

### 2.2. Experimental Groups

In order to explore the different conditions that contribute to developing WKS, the following experimental groups were used.

- Chronic alcohol (CA)

A protocol based on the work of Fernandez et al. (2016) [23] was employed. The ethanol solution was prepared from ethanol 96° (Iberalcohol S.L., Madrid, Spain) in tap water to the appropriate *v*/*v*. All rats had limited access to a single bottle, so the alcohol groups were exposed to forced consumption of an ethanol solution (20% *w*/*v*) as the sole source of liquid. Alcohol was gradually introduced using a “fading on” procedure; ethanol exposure started at 6% for 5 days, followed by another 5 days at 9%, 5 days at 12%, 2 days at 16% and finally reaching 20%, which was maintained during the 36-week duration of the experiment. CA rats (*n* = 9) were provided with standard food ad libitum throughout the treatment.

- TD diet (TDD)

Since the beginning of the study, the rats in the TDD group *(n* = 9) had access to a single bottle with tap water and were fed with the standard food, but in the last 12 days of the experiment, the chow was replaced by a thiamine-deficient diet (residual thiamine level in diet <0.5 ppm; Teklad Custom Diet, Envigo, Madison, WI, USA), in conjunction with daily pyrithiamine hydrobromide (thiamine pyrophosphokinase inhibitor) injections (Sigma Aldrich, Madrid, Spain; 0.25 mg/kg; i.p.) (WKS Pre-Symptomatic Model (PSM) protocol in [18]).

- Chronic alcohol combined with TD diet in the last days of treatment (CA+TDD)

The rats received the same alcohol treatment as the CA group, and just like the TDD group, in the last 12 days of the experiment, the standard food was replaced by the thiamine-deficient diet in conjunction with daily pyrithiamine hydrobromide injections (0.25 mg/kg; i.p.) (*n* = 10).

Furthermore, we included three control groups to monitor the alcohol and TD variables.

- Chronic alcohol with oral thiamine supplementation (CA+T)

We included a group receiving chronic alcohol with thiamine supplementation to deduce whether the effects of long-term alcohol exposure can be ameliorated with prophylactic treatment (*n* = 8).

Since it is a chronic supplementation with thiamine, the route of administration chosen was oral to avoid stress and discomfort of the animals produced by another type of procedure, such as daily injections for long periods of time. The pharmacological dose of thiamine hydrochloride (Sigma Aldrich, Madrid, Spain) chosen was 0.2 g/L, taking as reference the ethanol exposure studies [24,25,26,27,28]. The drink containing 20% (*w*/*v*) of alcohol was mixed with the thiamine as the sole drinking fluid for the 36 weeks of the experiment.

- Drinking water with oral thiamine supplementation (C+T)

Appropriate control subjects also received the same dose of 0.2 g/L of thiamine in the water throughout the experiment (*n* = 6).

Since no significant changes were found in any of the variables analyzed, the data corresponding to the CA+T and C+T groups are not shown in the results, as will be explained later.

- Drinking water with standard chow (C)

The rats in the control group had access to a single bottle with tap water and standard chow *ad libitum* for the entire duration of the study *(n* = 8).

#### Experimental Procedures

During the last 12 days of TDD protocol, the remaining animals (CA, CA+T, C+T and C) received equivalent daily injections of saline (i.p.) to reproduce the same stress conditions in all animals.

The body weights of all animals were recorded weekly (every Monday, Wednesday and Friday), except during the final TDD protocol, where the TDD and CA+TDD rats were weighed daily. Likewise, TD diet intake was also measured for these groups on those 12 days.

The volume of the bottles was measured and replenished every Monday, Wednesday and Friday. Additional bottles with the ethanol solution and tap water were included to control for spillage and evaporation throughout the experiment. The grams of ethanol per kilogram of body weight was determined by the following formula: [milliliters of ethanol consumed (loss control subtracted)] × the percent concentration of ethanol × the density of ethanol/weight of the animal. The average daily ethanol consumption was determined by dividing the weekly consumption by 7 days across the ethanol exposure phase.

### 2.3. Blood Ethanol Concentrations (BECs) and Thiamine Measurements

During the chronic alcohol protocol, initial, intermediate and final measurements of BEC and thiamine were performed in all animals. Blood samples were collected from the tail in weeks 4 and 12 of the experiment, and the final measurements were performed from trunk blood. Heparin (5% *w*/*v*) was used as an anticoagulant, and plasma was obtained by blood centrifugation.

The ethanol levels were determined by using electrochemical detection of an enzymatic reaction with an AM1 Alcohol Analyzer (Analox Instruments, London, UK) and also by using the EnzyChrom™ Ethanol Assay Kit (BioAssay Systems, Hayward, CA, USA, ECET-100) according to the manufacturer’s protocol.

Thiamine was measured using an Enzyme-Linked Immunosorbent Assay (ELISA) Kit for Vitamin B1 (VB1) (Cloud-Clone Corp.), following the manufacturer’s instructions.

### 2.4. Behavioral Assessment

**Figure 1:**
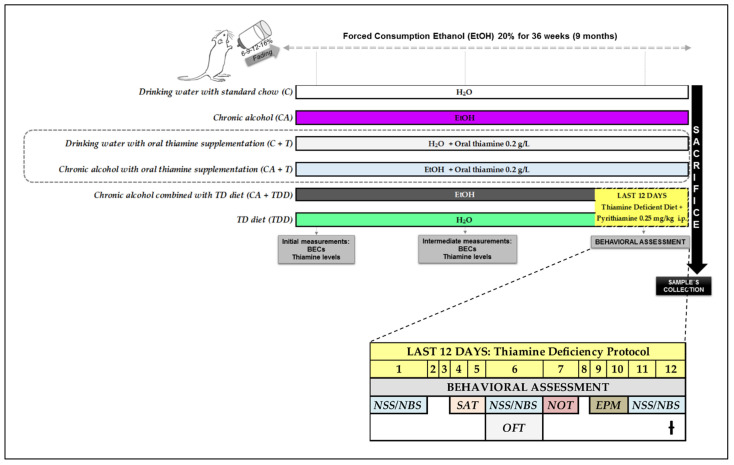
Experimental design, with timeline of treatments and behavioral testing. Body weight and water (H_2_O) and ethanol (EtOH) consumption were recorded throughout the entire experiment and the TD diet intake during the last 12 days. *BECs = blood ethanol concentrations; NSS/NBS = neurological examination; SAT* = spontaneous alternation task*; OFT* = open field test*; NOT* = novel object recognition test*; EPM*: elevated plus maze.

We focused on evaluation of the motor and cognitive domains, as we did in previous studies on WKS [18]. The time course of behavioral assessments, appropriately scheduled not to interfere between them, is depicted in Figure 1. Behavioral tests were performed at least 1 h after any pharmacological treatment and were separated by a sufficient time interval to avoid interferences. The animals were evaluated following an alternation of experimental groups in all tests. All apparatuses were carefully cleaned with ethanol (15% *w*/*v*) between trials to remove possible odor cues. Analyses of the behavioral test that were not computerized were performed by a double-blind protocol to ensure the reliability of the results.

- Neurological assessment

Motor and sensorial affectations, reflexes, walking and exploratory behavior in the home cage were evaluated by using the neurological severity (NSS) and neurobehavioral (NBS) scores according to our previous studies [18]. This evaluation was carried out especially for the possible effects of the TD diet treatment. Therefore, all the animals were monitored on day 1, 6 and 12 of the TDD protocol.

- Motor function: locomotion

The *open field test (OFT)* provides simultaneous measurement of locomotion and disinhibition-like behavior [29]. The apparatus is a square black box (80 × 80 × 42 cm) with the base divided into 6 × 6 cm equal squares with legible white lines. Each rat was placed at the corner of the floor at the beginning of the trial, allowing the animal to freely move and explore the platform surface for 5 min. Specific behavior parameters were registered using an automatic monitoring system (Smart software v. 2.0.14, Panlab). The general locomotor activity is represented by the total distance traveled (cm) and the walking speed (cm/s).

The *spontaneous alternation task (SAT)* also evaluated locomotor activity. The test was carried out in a symmetrical Y-shaped maze constructed of opaque black polyvinyl chloride (PVC). Each arm (40 × 15 × 30 cm) forms an angle of 120° with the next, and they are equipped with visual signs on the walls. The testing room was dimly lit by a white lamp with a luminosity between 15 and 17 lux. Each rat was allowed to freely explore the 3 arms of the maze for 8 min, and the trials were video recorded. The total number of arm entries was registered, considering an arm entry whenever a rat entered the arm with their 4 paws.

- Cognitive domain: disinhibition-like behavior and memory

We also used the OFT to evaluate disinhibition. The open field exposure paradigm is based on a conflict between the internal drive to explore a novel environment (based on the potential for rewarding outcomes) versus the internal drive to avoid a novel environment (based on the potential for aversive outcomes). Behavior in the OFT is influenced by various factors, including the level of illumination of the open-field arena. Bright light can be used as an aversive stimulus in the open-field paradigm, leading to an increase in avoidance behaviors. Rodents are nocturnal and tend to avoid brightly lit places. Here, we performed the test under white light conditions (50–60 lux). In addition, rodents also avoid wide open spaces and instead remain close to vertical references such as walls [30]. The platform surface was virtually divided into 2 regions: the center (inner zone) and the edges (in contact with the walls). Disinhibited rats showed a lack of environmental awareness by spending more time in the arena’s center. The percentages of time spent and distance traveled in the inner zone compared with the total have been represented.

The *elevated plus maze test (EPM)* is probably the most popular animal model of anxiety [31]. This model is based on a fear and curiosity balance toward novelty. When the typical rat behavior with appropriate anxiety and general avoidance of open areas is altered, a disinhibited response can be observed [32,33]. The EPM is made of two black and grey plastic open arms (50 × 10 cm) and two perpendicular closed arms of equal length and width but with 50-cm high opaque walls. A central area of 10 cm^2^ connected all arms. The platform was elevated 65 cm above the floor. When the rats are placed in this test apparatus, they exhibit a strong preference for the closed arms, indicating a fear reaction toward the open arms. This reaction is interpreted as accounting for anxiety [31]. Several factors may also influence the behavioral baseline (i.e., the open/total arm entries ratio), with the ambient light intensity being among the most effective ones. Increasing the ambient illumination induces increased anxiety responses in the EPM. Here, we manipulated the intensity of the anxiogenic stimuli by exposing them to conditions of white light of 55 lux. The homogeneity of the light intensity was settled at the center of the maze and at the extremity of the 4 arms by using a luxmeter. The maximal difference measured between all parts of the maze never exceeded 5 lux. At the beginning of a test, each rat was placed on the central platform facing a closed arm and opposite to the experimenter position. Then, the animal was allowed to explore the maze freely for 5 min. The number of entries and time spent in the open arms and the total arms (open and closed arms) were determined by a computer-controlled system recording the interruptions of infrared photo beams located along each arm. An entry in a given arm was counted when the rat had completely entered the arm (4 paws and tail in the arm). Data were analyzed by using the MAZEsoft software (Panlab, Barcelona, Spain).

The SAT also evaluated spatial working memory based on a natural tendency of rats to explore the less recently visited Y-maze arm [34]. The spontaneous alternation (successive entries into the 3 arms in overlapping triplet sets) index was calculated as the ratio of actual to possible alternations ((number of actual alternations/[total number of arm entries-2]) × 100) [35].

The *novel object recognition test (NOT)* analyzed possible impairments in the short-term recognition memory. The task was performed in a square arena (80 × 80 × 42 cm) with black matte-painted walls and a bright black wooden floor divided by white painted lines into 25 squares (6 × 6 cm). The arena was subdivided into 4 equal sections, allowing the evaluation of 3–4 animals simultaneously. The NOT was carried out in accordance with previous studies [35] with some minor modifications. The test was performed in low-light conditions (white light, 20 lux) and organized in 3 phases: habituation (time = 0), a training phase (pre-test) and a test session 4 h after the training phase. During the habituation period, the animals were allowed to freely explore the arena (without objects) for 5 min. In the training phase, 2 identical objects (glass bottles and the familiar objects (F) in the test session) were located in opposite corners of the arena, and the rats were allowed to freely explore them for 3 min. In the test session 4 h after the training phase, one of the familiar objects (F) was replaced by a novel object (N1, green ashtray), and the animals were allowed to explore both objects for 5 min. The objects with similar dimensions were selected, and their positions in the arena were alternated in order to avoid possible place preferences. At the beginning of each session, the animals were placed in the corner of the apparatus facing the wall opposite the objects. Both the training and test sessions were video recorded (Sony DCRDVD310E, Spain). Exploration of an object was considered whenever animals pointed their nose toward an object at a distance ≤1 cm, while turning around, climbing or biting the objects was not considered exploration. The time that the animals spent exploring the objects during the test sessions, as well as the latency to first explore the novel object, were registered. The discrimination index (DI) was calculated as the difference between the time spent exploring the novel object (N) and the familiar one (F1 or F2) in relation to the total time spent exploring the objects ((N − F)/(N + F)). Negative values of the discrimination index were considered null.

### 2.5. Blood and Tissue Sample Collection

On day 12 of the TDD protocol, at least 1 h after treatment administration, the animals were decapitated after lethal injection of sodium pentobarbital (320 mg/kg, i.p., Dolethal^®^, Vétoquinol, Spain). Blood for plasma determinations was collected from the trunk with tubes containing heparin (5% *w*/*v*, 1 vol heparin per 9 vol blood) as an anticoagulant. Plasma was obtained from blood samples by centrifuging at 1000 g for 15 min at 4 °C. All plasma samples were stored at −80 °C until assayed. The brains were immediately isolated from the skull, discarding the meninges and blood vessels, and the frontal cortex (area between the bregma +4.7 and +1.2 mm, approximately) was excised and frozen at −80 °C until assayed.

### 2.6. Protein Assay

Protein levels were measured by Bradford’s method (Bradford 1976) based on the principle of protein dye binding.

### 2.7. Western Blot Analysis

The frontal cortex samples were homogenized by sonication in PBS (pH = 7.4) and mixed with a protease inhibitor cocktail (Complete, Roche^®^, Madrid, Spain) in a dilution of 1:3 (*w*/*v*), followed by centrifugation at 13,000 rpm for 10 min at 4 °C. After adjusting the protein levels, the homogenates were mixed with Laemmli simple buffer (Bio-Rad^®^, Alcobendas, Madrid, Spain) containing β-mercaptoethanol (50 μL/mL of Laemmli) to obtain a final concentration of 1 mg/mL, and 15–20 uL was loaded into an electrophoresis gel. The proteins were blotted onto a nitrocellulose membrane (Amersham Iberica^®^, Madrid, Spain) with a semi-dry transfer system (Bio-Rad), incubated with specific primary and secondary antibodies (see Table 1) and revealed by using an ECL™ kit (Amersham Iberica, Madrid, Spain). Autoradiographs were quantified by densitometry (NIH ImageJ^®^ software, National Biosciences, Lincoln, NE, USA) and expressed as the optical density (O.D.). In all western blot analyses, the housekeeping β-actin protein was used as a loading control.

Each blot contained different biological replicates per group, and 3 blots were run in separate assays. The results represent the average of the 3 technical replicates. The blot images above the results graphs were always taken from the same gel. The samples from all experimental groups were always loaded randomly. For this reason, and in order to follow the same order of group presentation in the graphs along the manuscript (and excluding groups C+T and CA+T), some bands were spliced in the figures.

### 2.8. Plasma Nitrites (NO_2_^−^) Levels

As the stable metabolites of the free radical nitric oxide (NO·), NO_2_^−^ was measured by using the Griess method [36]. In an acidic solution with 1% sulphanilamide and 0.1% N-(1-Naphthyl) ethylenediamine (NEDA), the nitrites converted into a pink compound that was photometrically calculated at 540 nm in a microplate reader. The results were expressed as percentage of the control.

### 2.9. Statistical Analysis

Data, expressed as mean ± S.E.M, were analyzed by two-way ANOVA (with repeated measures for weight and consumption data) when normality was verified; otherwise, a Kruskal–Wallis test was used. Homocedasticity was checked by Barlett’s test and data transformed (sqrt) when appropriate. In the ANOVA, Bonferroni post hoc tests were used when appropriate. The outliers were analyzed using Grubbs’ test. Correlations between the behavioral and biological measures were assessed by Pearson’s and linear regression analyses. To account for multiple testing in the correlations, we adjusted the *p*-values by controlling the false discovery rate (FDR), which was set to 0.05 and assessed with the Benjamini–Hochberg procedure. This type of correction allows for restricting the occurrence of false-positive findings (type I error) among all nominal significant findings. A *p* value <0.05 was set as the threshold for statistical significance in all statistical analyses. The data were analyzed using GraphPad Prism version 8.0 (GraphPad Software, Inc., La Jolla, CA, USA).

## 3. Results

### 3.1. Weight and Consumptions

#### 3.1.1. Body Weight throughout the 36 Weeks of the Experiment and TD Diet Intake

**Figure 2:**
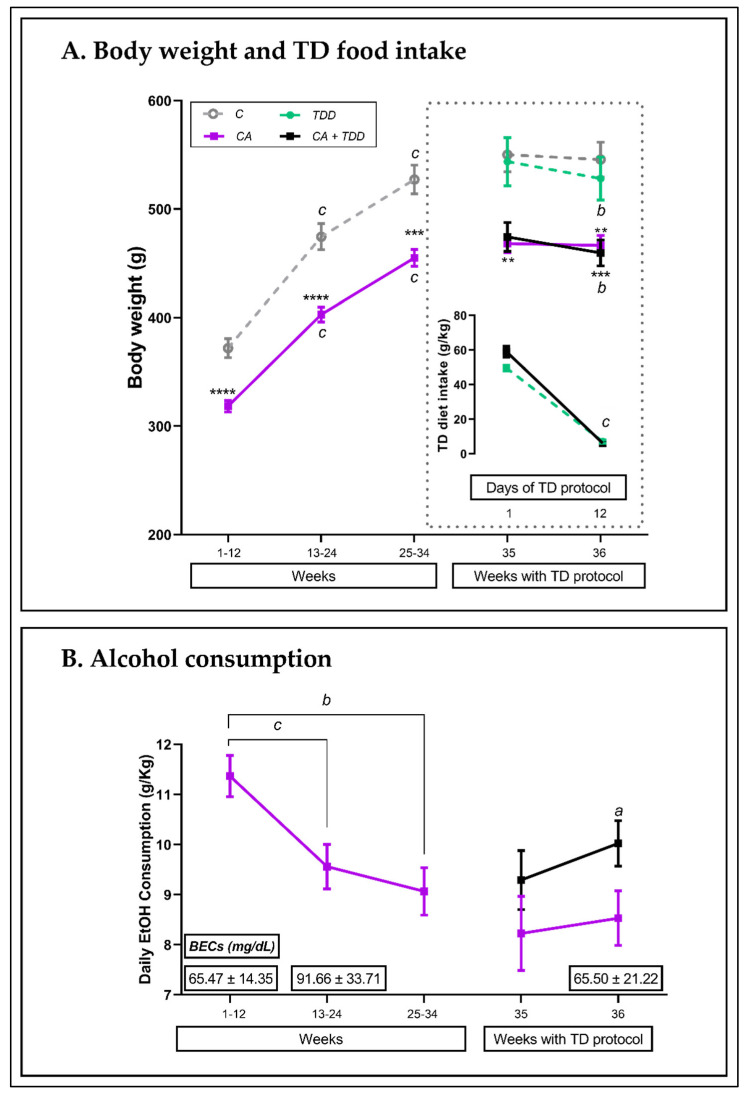
Physiological parameters along the procedure. (**A**) Average weights at weekly intervals and at days 1 and 12 of the final TD diet protocol, along with the diet intake (g/Kg). All rats gained weight until week 34, but the CA animals were always below the controls. Weight loss in TDD and CA+TDD groups during TD diet treatment was consistent with the decreased intake. (**B**) Ethanol consumption (g/Kg) across weeks, along with blood ethanol concentrations (BECs (boxed numbers): mean ± SEM). The animals consumed more alcohol during their adolescent and young stages than in the adult stage. All data are expressed as mean ± S.E.M. Repeated measures ANOVAs; within–group differences: *a p* < 0.05; *b p* < 0.001; *c p* < 0.0001; between–group (different vs control group): ** *p* < 0.01; *** *p* < 0.001; **** *p* < 0.0001.

All the rats gained weight during the course of the experiment (Figure 2A; two-way repeated measures (RM) ANOVA; interaction between factors (time × treatment), F _(2, 68)_ = 7.006, *p* = 0.0017; main effects of time: F _(1,122, 38,15)_ = 1360, *p* < 0.0001 and treatment: F _(1, 34)_ = 27.43, *p* < 0.0001; within-group differences, *p* < 0.0001), but the CA animals weighed significantly less than the control subjects during all weeks (between-group differences, CA vs. C weeks 1–12 and 13–24: *p* < 0.0001; weeks 25–34: *p* = 0.0002).

With the start of the TD diet, the four corresponding groups were fully shaped, and significant weight loss was observed in the TDD and CA+TDD animals at the end of the protocol (Figure 2A, upper right box; two-way RM ANOVA, interaction: F _(3, 32)_ = 4.742, *p* = 0.0076; effect of time: F _(1, 32)_ = 30.24, *p* < 0.0001; effect of treatment: F _(3, 32)_ = 8.26, *p* = 0.0003; both groups: *p* = 0.0002).

This weight loss in the TDD and CA+TDD groups was consistent with a decrease in their food intake (Figure 2A, lower right box; two-way RM ANOVA, interaction: F _(1, 17)_ = 8.932, *p* = 0.0083; effect of time: F _(1, 17)_ = 745,8, *p* < 0.0001; both groups: *p* < 0.0001).

#### 3.1.2. Alcohol Intake and BECs

As shown in Figure 2B, there were differences in the amount of alcohol consumed across treatments (RM one-way ANOVA, F _(1,259, 22,67)_ = 15.54, *p* = 0.0003). In the early adolescent stages and younger, the rats drank more than during the adult stages (weeks 13–24 vs. 1–12: *p* < 0.0001; weeks 25–34 vs. 1–12: *p* = 0.0001).

During TDD treatment, the CA+TDD rats displayed an increase in ethanol consumption between the end and beginning of TD diet treatment (week 36 vs. 35) (two-way RM ANOVA, overall effect of time, F _(1, 17)_ = 5.859, *p* = 0.027; *p* = 0.0478), which was not observed in the CA group (*p* > 0.05, n.s.). No significant differences were found between the CA groups with and without the TD diet (*p* > 0.05, n.s).

The blood ethanol levels (indicated in Figure 2B with boxed numbers), measured at 1 month, 3 months and 9 months of CA (weeks 4, 12 and 36), did not change significantly across the months (F _(2, 17)_ = 0.4223, *p* > 0.05, n.s). The global BEC mean was 73.33 ± 12.65 mg/dL, modeling moderate drinking. Samples from some randomized control animals were also measured as technical controls, obtaining values ranging from 3 to 7 mg/dL.

### 3.2. Neurological Examination

No significant neurological signs or behavioral disturbances were found in animals through NSS/NBS examination on days 1, 6 and 12 of the TDD protocol.

We explored all rats in the open surface and in their home cages, checking the general activity, flexion of limbs, extension of the head, movements of the tail, posture and reflexes, among other factors. Only some slight deficit in the startle reflex in a few alcohol-treated animals (CA and CA+TDD) was found, which seemed to not react to the sound stimulus (claps or finger snaps) as controls. Beyond this, no other symptoms or evidence of motor alteration, such as ataxia, were detected due to the treatments.

### 3.3. Behavioral Outcomes

#### 3.3.1. Effects on Locomotor Activity and Spatial Memory: SAT

**Figure 3:**
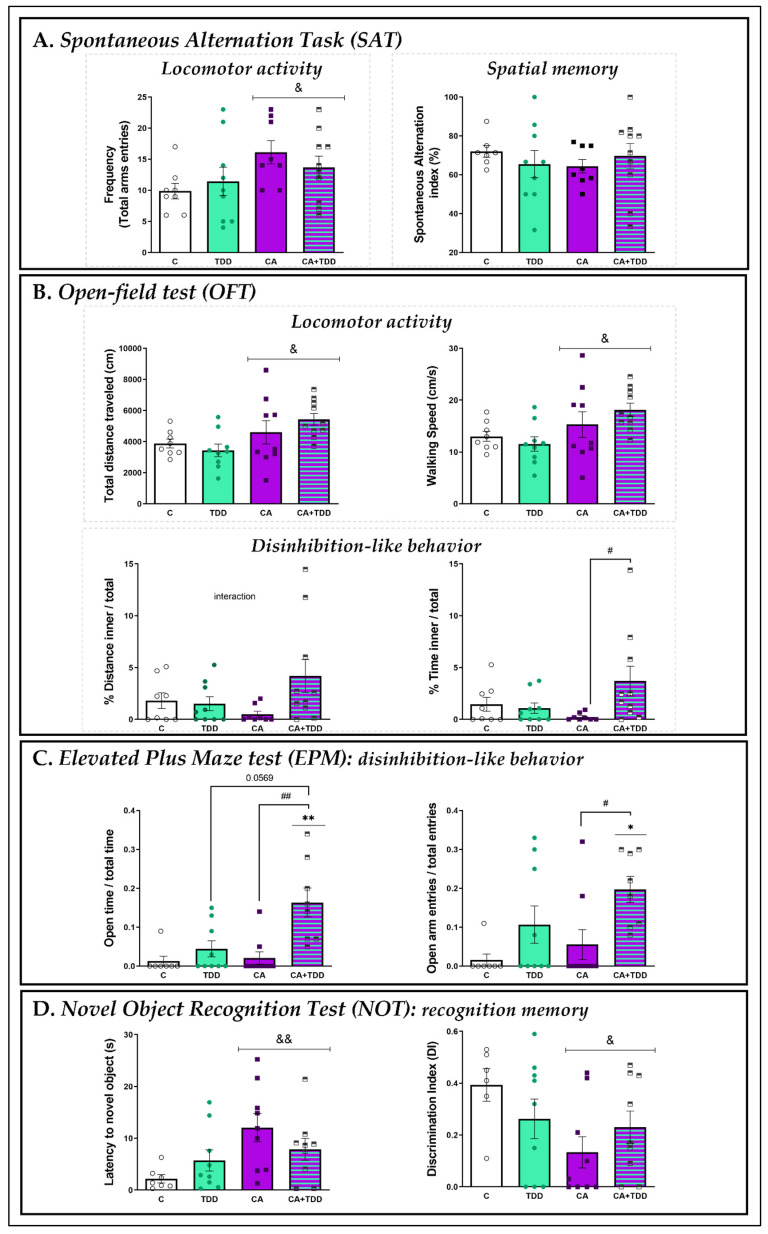
Behavioral changes as a function of the treatment condition. (**A**) Spontaneous alternation task (SAT). There was an increase in the total arm entries by the CA groups independent of TDD treatment, interpreted as hyperlocomotion, and no effect on spontaneous alternation reflecting spatial working memory. (**B**) Open field testing (OFT) locomotion and disinhibition. Here, we also found motor agitation by alcohol. Rats typically avoid open areas. CA+TDD-treated rats explored the inner zone for more time than the rest of group, reflecting a trend of disinhibition. (**C**) Elevated plus maze (EPM). Time spent and entries in open arms showed a remarkable disinhibited behavior just in rats with CA combined with TDD treatment. (**D**) Effect on memory abilities tested by NOT. Rats exposed to CA were impaired in their ability to discriminate the new object, thus showing a deficit in recognition memory. Mean ± SEM (*n* = 8–10). Two-way ANOVA or nonparametric Kruskal–Wallis test. Overall alcohol effect: ^&^ *p* < 0.05, ^&&^ *p* < 0.01. Interaction (CA × TDD) followed by post hoc test, different from control group: * *p* < 0.05, ** *p* < 0.01; different from CA: ^#^ *p* < 0.05, ^##^ *p* < 0.01.

Two-way ANOVA revealed a main stimulatory effect of chronic alcohol on locomotor activity, since the number of total arm entries of the CA and CA+TDD rats was significantly increased (Figure 3A, left graph, overall effect of alcohol F _(1, 31)_ = 5.152, *p* = 0.0303).

No effects on spontaneous alternation were observed due to alcohol or TDD, nor was there a significant interaction between these factors. Thus, spatial working memory did not appear to be affected by the treatments (Figure 3A, right graph, *p* > 0.05, n.s.).

#### 3.3.2. Effects on Locomotor Activity and Disinhibition-Like Behavior: OFT

Like in the SAT, a significant overall effect of alcohol was found on OFT locomotor activity, increasing the total distance traveled and walking speed in the CA and CA+TDD animals (Figure 3B, upper left graph, F _(1, 32)_ = 6.859, *p* = 0.0134; upper right graph, F _(1, 32)_ = 7.348, *p* = 0.0107, respectively).

In addition, there was an interaction between CA and TDD in the percentage of the distance travelled and time spent in the internal zone of the field (Figure 3B, lower left graph, F _(1, 31)_ = 4.246, *p* = 0.0478; lower right graph, F _(1, 31)_ = 5.444, *p* = 0.0263, respectively). Post hoc comparisons revealed a trend for the CA+TDD group to walk more in the inner zone (*p* = 0.066) and a significant effect of the time spent in the inner zone with respect to the CA rats (*p* = 0.028). In this way, the rats treated by alcohol combined with TDD showed the highest percentages among all groups.

Thus, the CA+TDD rats appeared to be disinhibited by preferring the internal area of the arena, which is known to be a more anxiogenic zone than the rest of the arena.

#### 3.3.3. Effects on Disinhibition-Like Behavior: EPM

As shown in Figure 3C, the animals exposed to CA along with the TDD displayed a higher percentage of time spent in the open arms compared with the controls (*p* = 0.0036) as well as versus the CA group (*p* = 0.0039), and the comparison with the TDD group was very close to the significance (*p* = 0.0569) (left graph, Kruskal–Wallis = 15.74, *p* = 0.0013).

Likewise, the CA+TDD rats also explored the open arms significantly more than the control (*p* = 0.0151) and CA (*p* = 0.0441) animals, as indicated by the percentage of entries (right graph, Kruskal–Wallis = 11, *p* = 0.0117).

Accordingly, similar to what was observed in the OFT, the combination of both treatments (CA+TDD) exclusively had a significant effect on disinhibition.

#### 3.3.4. Effects on Recognition Memory: NOT

For the training phase (pre-test), we found no differences between the animals in their reaction to unfamiliar objects. CA+TDD rats did not exhibit significantly higher inclinations to explore novel objects (neither lower latency nor greater exploration time) compared with the other groups (latency (s): C = 24.1 ± 7, TD = 23.4 ± 5.2, CA = 35.7 ± 6.5, CA+TDD = 17.2 ± 2.6; exploration time: C = 24.4 ± 2.6, TD = 21.1 ± 2.1, CA = 18.4 ± 2.7, CA+TDD = 24.7 ± 2, *p* > 0.05 n.s.). There were also no significant differences in the total time that animals spent exploring the objects during the test session (C = 43.1 ± 4.1; TD = 43.3 ± 6; CA = 45.8 ± 5; CA+TDD = 45.7 ± 3.3; *p* > 0.05 n.s.).

However, during the test session, the time of first approach to the novel object rendered a general significant effect of alcohol, so the CA and CA+TDD animals displayed higher latency (Figure 3D, left graph, F _(1, 30)_ = 7.652, *p* = 0.0096).

Furthermore, the two-way ANOVA of the discrimination index data also demonstrated a significant alcohol effect, decreasing their recognition capacity (Figure 3D, right graph, F _(1, 29)_ = 4.557, *p* = 0.0414).

Therefore, chronic exposure to alcohol seemed to cause a cognitive impairment by worsening the memory capabilities of the CA and CA+TDD animals.

### 3.4. Alterations Found in the Frontal Cortex and Plasma

#### 3.4.1. Effects on Oxidative and Nitrosative Stress and Lipid Peroxidation Markers

We measured the markers that led to nitrosative stress, such as the inducible nitric oxide synthase (iNOS) enzyme and the accumulation of the stable metabolite of NO, NO^−^_2_.

**Figure 4:**
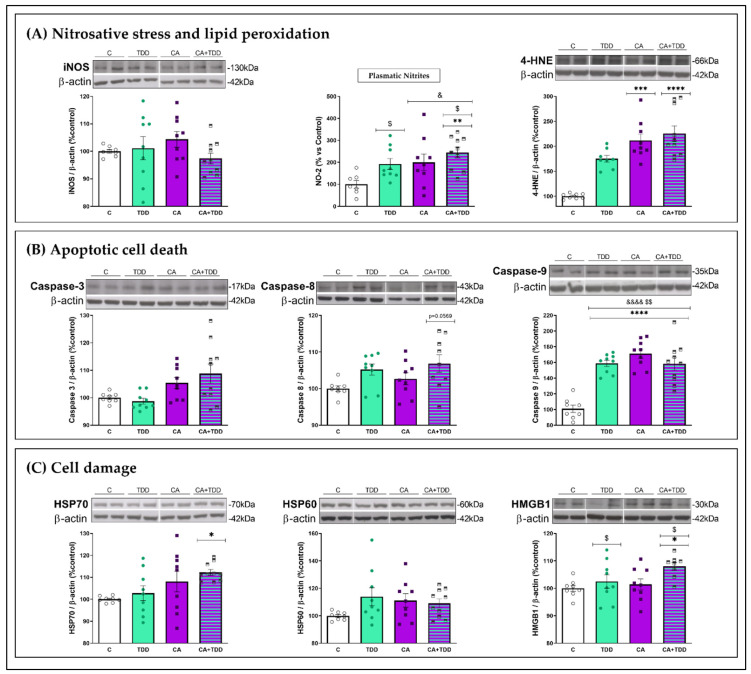
Biochemical changes as a function of treatment condition. Graphs indicate protein levels of markers in the frontal cortex and plasmatic nitrites. Western blot data of the respective bands of interest (upper bands) were normalized by β–actin (lower band) and expressed as a percentage of change versus the control group. (**A**) Nitrosative stress markers. Nitrites (NO−_2_) increased in plasma for all conditions of exposure, but the combination of CA and TDD enhanced this elevation. Lipid peroxidation was measured by 4–Hydroxynonenal (4–HNE) accumulation. There was a significant effect of CA and CA+TDD groups versus control group. (**B**) Markers of apoptotic cell death: caspase 3, 8 and 9. All treatments led to an elevation of caspase 9 relative to the controls. (**C**) Damage-associated molecular patterns (DAMPS): HSP70, HSP60 and HMGB1. CA+TDD treatment increased the HSP70 and HMGB1 expression with respect to the controls. Mean ± SEM (*n* = 8–10). Two-way ANOVA or nonparametric Kruskal–Wallis test. Overall alcohol effect: ^&^ *p* < 0.05, ^&&&&^ *p* < 0.0001; overall TDD effect: ^$^ *p* < 0.05, ^$$^ *p* < 0.01; and interaction (CA × TDD) followed by post hoc test, different from control group: * *p* < 0.05, ** *p* < 0.01, *** *p* < 0.001, and **** *p* < 0.0001.

The protein levels of iNOS enzyme were analyzed in the frontal cortex, and no changes were detected (Figure 4A, *p* > 0.05 n.s.). Nonetheless, the nitrite (NO^−2^) levels were studied at the peripheral level (Figure 4A). The analysis of the plasma showed the main effects of alcohol (F (1, 31) = 7.377, *p* = 0.0107) and TDD treatment (F (1, 31) = 5.924, *p* = 0.0209). Post hoc comparisons revealed that the nitrites level were significantly higher in the CA+TDD group compared with the controls (*p* = 0.0073).

These results indicate that CA consumption interacted with the TDD, inducing a notable increase in the markers of peripheral nitrosative stress.

Lipid peroxidation is a consequence of excessive oxidative and nitrosative cellular stress. We measured 4-hydroxynonenal (4-HNE) as an aldehydic product of lipid peroxidation. As shown in Figure 4A, rats exposed to CA showed a significant increase in the 4-HNE protein levels compared with the controls (*p* = 0.0006), with this effect being even more pronounced by the combined treatment with the TDD (*p* < 0.0001) (Kruskal–Wallis = 22.72, *p* <0.0001).

Thus, our results show that CA and CA+TDD treatments produce damage to the cellular lipid membranes in the frontal cortex cells.

#### 3.4.2. Effects on Apoptotic Markers

Different caspases, such as 3, 8 and 9, could generate apoptotic cell death.

With respect to caspase 3 and caspase 8, there were significant differences between groups (Figure 4B, Kruskal–Wallis = 9.062; 8.04, *p* = 0.0285; 0.0452, respectively). In particular, the group with the combination of CA and TDD treatments showed a trend to the highest levels of expression of these caspases, with levels very close to the significance for caspase 8 (*p* = 0.0569) compared with the control group.

In relation to caspase 9, an interaction between factors was found (F _(1, 32)_ = 32.45, *p* < 0.0001), together with the main effects of alcohol (F _(1, 32)_ = 31.49, *p* < 0.0001) and TDD treatment (F _(1, 32)_ = 13.09, *p* = 0.001). Post hoc analysis revealed higher levels in all treated groups compared with the control group (Figure 4B; for all comparisons, *p* < 0.0001).

#### 3.4.3. Effects on Cell Damage Markers

Some molecules can be released in response to injured tissue, such as heat shock proteins (HSPs) and the high-mobility group box 1 protein (HMGB1).

We found significant changes in the HSP70 levels (Figure 4C, Kruskal–Wallis = 8.874, *p* = 0.031) only in the CA+TDD group, which were significantly higher than those in the control group (*p* = 0.0325). There were no differences in the HSP60 expression levels between the different experimental groups (Figure 4C, *p* > 0.05 n.s.).

Regarding HMGB1 protein expression, there was a main effect (upregulation) of the TDD (Figure 4C, F _(1, 30)_ = 5.905, *p* = 0.0213). Post hoc analysis also showed that the increase was greater in the CA+TDD group relative to the controls (*p* = 0.0377).

### 3.5. Correlations and Linear Regression Analyses between Biochemical and Behavioral Parameters

Here, we hypothesized that the signals of damage found in the frontal cortex for the CA+TDD animals were, to some extent, related to the behavioral alterations observed. Exclusively in this model with the combination of both treatments, we found disinhibition as the clearest alteration in behavior. This dysfunction may reflect a deficit in executive functioning that has been related to frontal brain damage in WKS patients [9].

**Figure 5:**
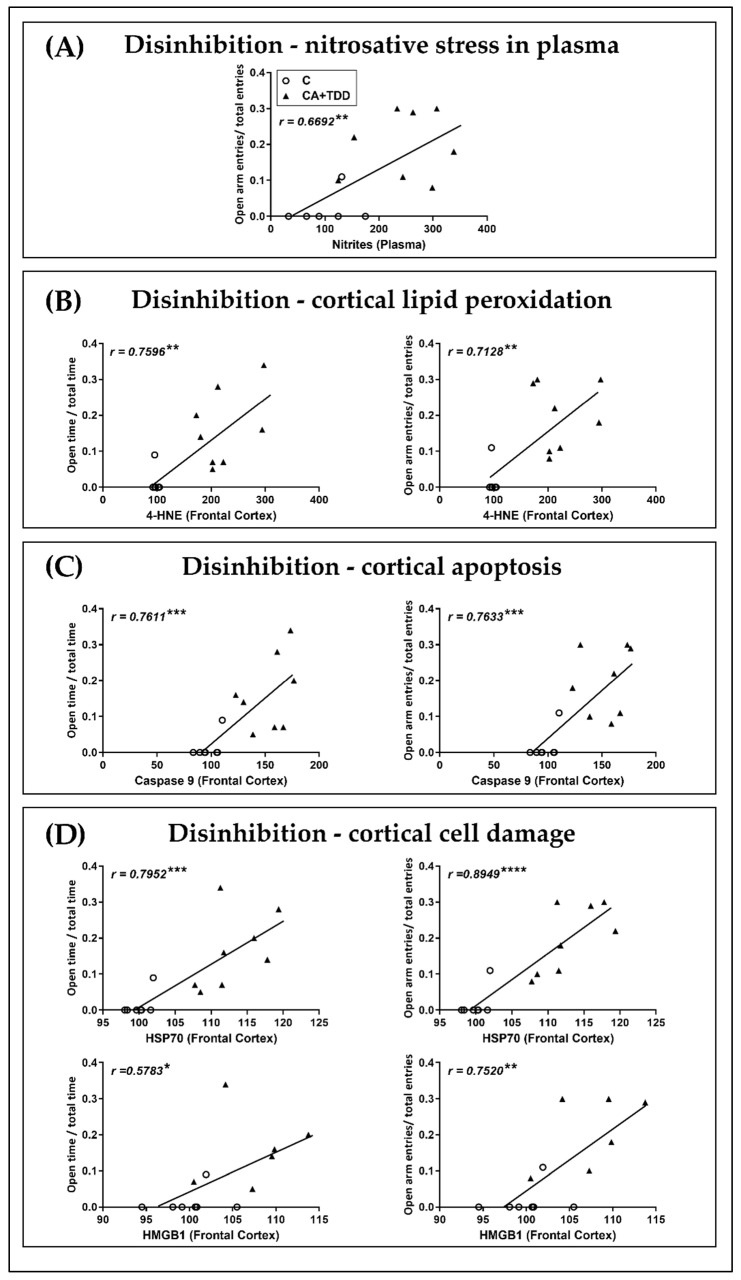
Correlations between damage parameters and disinhibition measures of the EPM in the CA+TDD-treated animals. The trend lines on each graph show the combined regression analyses for the control (circles) and CA+TDD (triangles) groups. (**A**) NO^−2^ levels in plasma were positively correlated with the EPM open entries ratio. Frontal cortical levels of (**B**) 4-HNE, (**C**) Caspase 9 and (**D**) HSP70, HMGB1 were positively correlated with the EPM open time and entry ratios. Pearson’s coefficient correlation *r;* * *p* < 0.05, ** *p* < 0.01, *** *p* < 0.001 and **** *p* < 0.0001.

Then, considering the most noteworthy biochemical changes and behavioral outcomes in our combined model, we performed correlation analyses that are summarized in Table 2. Additionally, an FDR adjustment to avoid type I errors in multiple correlations was performed. Here, 90% (9 of 10 total) of the correlations were positive (Table 2) and passed the FDR adjustment at 5% (graphed in Figure 5).

We found that the NO^−2^ levels in the plasma were positively correlated with the disinhibition measures (Figure 5A).

The analyses also indicate a possible relationship between the cortical alterations and the disability in behavioral inhibition found. Specifically, elevations in the frontal cortex key parameters of lipid peroxidation (4-HNE), apoptosis (caspase 9) and cell damage (HSP70, HMGB1) positively correlated with the disinhibition-like behavior observed in the EPM (Figure 5B–D).

## 4. Discussion

Wernicke’s encephalopathy and Korsakoff ’s syndrome are different stages of the same disease, named WKS [37]. Taking into account the factors and conditions that contribute to developing WKS, we used several animal models to investigate different aspects within this disorder at different degrees or severities. In this study, WKS was modeled by CA consumption, since AUD is the main cause of this pathology, with and without a TD diet to understand the contribution of alcohol and the nutritional deficiency of thiamine to the pathology. Our results showed that CA affected the locomotion and memory, but only in conjunction with the TDD led to a pronounced disinhibition-like behavior. Likewise, the combination of both treatments led to the greatest alterations in the peripheral and cortical markers of oxidative and nitrosative stress and cellular and apoptotic damage. Such injury signals correlated with the inhibitory deficit, suggesting that the frontal cortex is a vulnerable brain area targeting the combined effects of CA and the TDD on cell viability and behavior.

In the present study, we observed a marked increase in the locomotor activity (SAT and OFT) as a consequence of CA treatment. These findings could be explained by the development of sensitization to the locomotor stimulant effects of alcohol [38]. This hyperlocomotion could not be conceptualized as a manifestation of behavioral motor disinhibition, since the CA animals were able to control and inhibit that motor activity, adjusting their behavioral responses to contextual elements. Indeed, hyperlocomotion and disinhibition would be different behavioral patterns mediated by different neural substrates. The sensitization in locomotor activity could reflect an increase in alcohol “wanting”, thus playing an important role in addiction [38]. Certainly, this phenomenon has been studied more in mice versus just a few works reported in rats [39,40,41]. Therefore, our study also extends these previous reports on rats.

In addition, alcohol-related neuropsychological impairments have been widely described in the literature, being executive functions, with working and episodic memory frequently affected [42]. Many experimental paradigms have been used to evaluate the memory deficits in alcohol-treated rats. However, several of these works show that depending on the treatment protocol, long-term alcohol intake does not necessarily lead to cognitive impairment [43]. In our research, a chronic pattern of alcohol consumption did not appear to affect the spatial memory of the animals (SAT). Nevertheless, controversial results have been reported following CA treatment, since Vedder et al. (2015) [44] also used the spontaneous alternation task and found deficits in the spatial memory of their animals. It is possible that the discrepancies in the results obtained under similar experimental conditions might depend on differences in the duration of alcohol treatment, BECs achieved, the presence or absence of a withdrawal period following ethanol administration or the age and strain of the rats, among other factors.

Regarding another kind of memory task analyzed, the CA animals exhibited a deficit in recognition memory (NOT), in line with previous research showing that alcohol intake impairs the performance of rats for the object recognition test [43,44,45].

Thus, our results appear to indicate that CA interferes with recognition memory but not with spatial memory. A possible interpretation of these findings could be that the mentioned tasks recruit different brain structures. Spatial memory performance is more dependent on the hippocampal function than recognition memory [46]. By contrast, recognition memory appears to be more dependent on cortical structures, such as the medial prefrontal cortex [47]. According to this, it has been demonstrated that the prefrontal cortex is more vulnerable to chronic alcohol-induced toxicity than the hippocampus [48]. Thus, recognition memory would be more sensitive to the cortical damage found in this study, whereas the hippocampus could be less affected. Further studies are needed to clarify this point.

In addition, the TDD alone did not affect any type of memory studied, nor was there any additive or synergistic interaction between CA and TDD in this regard. In contrast, we found that exclusively CA combined with TDD induced a pronounced disinhibition-like behavior (EPM). The CA+TDD rats exhibited an increase in the percentage of entries and time spent in the open arms relative to the controls, which was interpreted as increased risk-taking behavior or impulsivity [32,33]. This effect is not observed by CA exposure alone, so this may indicate that the TDD is necessary to develop an impulsive or disinhibited behavior. In fact, we recently reported such disinhibitory behavior in a non-alcoholic WKS model based on a TD diet, where TD-induced brain damage was exacerbated by glucose loading [18]. Furthermore, we also found this disinhibitory effect in the OFT, since the CA+TDD animals walked and spent more time in the arena center, in contrast to a typical rat’s behavior, showing general avoidance of open areas [29]. Indeed, although the OFT and EPM are usually considered models of anxiety-like behaviors, growing evidence also supports these alternative interpretations [29,32,33]. These responses of the CA+TDD animals in the EPM and OFT may signify poor impulse control and higher drive toward risk-taking behaviors due to a lack of environmental awareness. Their inclination to walk on a novel, flexible bridge elevated 65 cm above the floor appears to reflect a lower assessment of potential or actual dangers. These key features may indicate potential impairments in inhibitory control processes, thus suggesting a predominantly disinhibitory effect. Nevertheless, a possible link between disinhibition and novelty- and sensation-seeking behaviors has been reported [49], which can be measured in rodents as a function of the exploratory activity toward unfamiliar environments and objects [50]. However, although the CA+TDD animals displayed disinhibition-like behavior, we did not find a novelty-seeking response that interfered with the NOT.

Disinhibition reflects a deficit in executive functioning, which is associated with frontal brain damage [10]. This has been revealed as one of the most important alterations suffered by WKS patients, with devastating emotional and social consequences [9]. Moreover, an impulse control deficit is associated with addiction [51].

The frontal cortex is responsible for executive functions and the inhibition of impulsive responses, and it is particularly sensitive to alcohol- and TD-induced damage [16,18,48,52]. In particular, it has been shown that alcohol and TD lead to upregulated expression of the iNOS enzyme [17,18,53]. In addition, iNOS can produce large amounts of NO that will react, forming reactive nitrogen species (RNS), which are responsible for oxidative and nitrosative stress [54]. These molecules carry out the cytotoxic process of lipid peroxidation, releasing toxic products from the degradation of membrane phospholipids such as 4-HNE and leading to cell death.

At the peripheral level, the results revealed that CA and the TDD individually increased the NO^−2^ levels in the plasma, with an additive effect in the NO^−^_2_ elevation exerted by the combination of both treatments. Due to the location of RNS production from different tissues, plasma nitrites can be a useful global indicator of the degree of damage. However, we found no significant changes in the iNOS levels in the frontal cortex, which can be attributed to the specific time point of sample collection. Nevertheless, CA exposure did increase the 4-HNE levels in the frontal cortex, observing, again, a greater increase with the combined treatment (CA with the TDD).

Consequently, ethanol-induced oxidative stress can be responsible for apoptosis and neuronal dysfunction (reviewed in [1,48]). In contrast, several studies showed that TD triggers more oxidative stress and severe cellular death than alcohol. In turn, ethanol and TD showed additive and synergistic toxicity in cellular death [55]. Recent studies indicate that TD can use the caspase 3 classical pathway to amplify apoptosis as alcohol intoxication, although both alcohol and TD can induce cellular death by different pathways (reviewed in [56]). Taking into account our data, these observations may depend on the pattern of alcohol consumption and the degree of TD. We found upregulation of caspases 3 and 8 in our previous studies with alcohol binge drinking as a precursor of the emergence of damage molecules such as HMGB1 [17]. Here, CA+TDD treatment did not modify the levels of caspase 3 or 8 in the frontal cortex, although a trend toward upregulation could be observed. Again, the time course of expression of such parameters may influence these results. However, caspase 9 appeared to be more susceptible to upregulation by all the treatments, showing no additive effect by the combined CA+TDD treatment, which could be due to a ceiling effect in the caspase expression.

In addition, alcohol and TD have been involved in the release of damage-associated molecular patterns (DAMPs) from cells in response to cellular stress or tissue injury [17,18]. DAMPs such as HSP60-70 and HMGB-1 active the innate immune system through TLR2 and TLR4 signaling. Thus, these molecules can lead to a vicious cycle by amplifying the neuroinflammation and inducing more cell damage (reviewed in [1]). Our findings showed that the combination of CA with the TDD induced an increase in the HSP70 and HMGB1 cortical levels. The TDD’s main effect of upregulating HMGB1 may suggest a greater influence of this treatment.

In light of our results, we can conclude that CA in conjunction with the TDD led to the most severe cortical, peripheral and behavioral alterations. In accordance, higher levels of cortical lipid peroxidation and apoptotic and cell damage markers, as well as peripheral NO^−^_2_, correlated with the open arms exploration in the EPM, suggesting a link between the cortical injury and the disinhibition-like behavior, a core symptom of the pathology, as was discussed above.

## 5. Conclusions

Our study modeled WKS through CA consumption, TD or the combination of both. The animal model based in CA consumption showed a sensitization to the hyperlocomotor effect of alcohol together with a recognition memory deficit but only in conjunction with the TDD led to disinhibition-like behavior, a core characteristic of the pathology. CA and the TDD individually caused selective cortical damage and an increase in NO^−2^ in the plasma, whereas the combination of these treatments aggravated the alterations in the markers of nitrosative stress, lipid peroxidation, apoptosis and cell damage studied. Thus, these processes appear to play a critical role in the cortical injury that could be related to the deficit in its inhibitory function. In this way, the nutritional deficiency appears to be implicated in the production of the disinhibited behavior, which is even more limiting in the daily life of WKS patients than memory problems.

Therefore, our study shed light on the underlying disease-specific mechanisms, reinforcing the need for neuroprotective therapeutic approaches with antioxidant and anti-apoptotic actions, along with preventive treatments of the nutritional deficiency in WKS.

## Figures and Tables

**Table 1:** Specific antibodies used in western blotting to detect proteins of interest.

Protein	Primary Antibody ^1^	Secondary Antibody
Lipid peroxidation marker	1:1000 BSA 3%	Mouse (1:2000)
4-HNE	MAB3249 R&D Systems	
Cell damage markers		
HSP70	1:1000 sc-1060	Goat (1:4000)
HSP60	1:1000 sc-13,966	Rabbit (1:2000)
HMGB1	1:1000 sc-56,698	Mouse (1:2000)
Apoptosis markers		
Caspase 3	1:500 sc-56,053	Mouse (1:2000)
Caspase 8	1:400 sc-81,656	Mouse (1:2000)
Caspase and pro-9	1:500 BSA 1% sc-56,076	Mouse (1:2000)
Nitrosative stress marker		
iNOS	1:500 BSA 2% sc-650	Rabbit (1:2000)
Housekeeping β-actin	1:10,000 A5441 Sigma	Mouse (1:10,000)

^1^ Dilutions and references (codes). Abbreviations: 4-HNE = 4-Hydroxynonenal; HSP70/HSP60 = heat shock protein 70/60; HMGB1 = high-mobility group box 1 protein; iNOS = inducible oxide nitric synthase; BSA = bovine serum albumin; sc = Santa Cruz Biotechnology. Sources of secondary antibodies: anti-mouse IgG, HRP-linked whole Ab (from sheep) (GE Healthcare, Chicago, IL, USA, ref. NXA931); anti-goat IgG (whole molecule), peroxidase antibody produced in rabbit (Sigma-Aldrich, St. Louis, MO, USA, ref. A5420); anti-rabbit IgG (H+L) cross-adsorbed secondary antibody, HRP conjugate (from donkey) (Thermo Fisher Scientific, Waltham, MA, USA, Ref. 31,458).

**Table 2:** Correlational, linear regression and FDR analyses between key damage markers and behavioral parameters in the CA+TDD model *(*Pearson’s coefficient correlation r; slope and constant from the linear regression analyses, *p-*value for both correlation and linear regression and *q*-value from FDR analyses). Corrected *p*-values found using the Benjamini–Hochberg procedure (FDR set at 0.05) to restrict the type I error in multiple comparisons. Significant correlations (*p* < 0.05) and FDR-corrected *p*-values (*q* < 0.05).

	Percentages in Open Arms in the Elevated Plus Maze ^1^									
	Time					Entries				
Protein Marker ^2^	r	Slope	Constant	*p*-Value	*q*-Value	r	Slope	Constant	*p*-Value	*q*-Value
Plasma NO^−2^	0.5044	0.0005792	−0.00688	0.0659	0.0659	0.6692	0.0008195	−0.03051	0.0089	0.0111
4-HNE	0.7596	0.001149	−0.09649	0.001	0.002	0.7128	0.001158	−0.07876	0.0029	0.0043
Caspase-9	0.7611	0.002541	−0.2306	0.001	0.002	0.7633	0.002739	−0.2364	0.0009	0.002
HSP70	0.7952	0.0118	−1.169	0.0004	0.002	0.8949	0.01427	−1.413	<0.0001	0.001
HMGB1	0.5783	0.01111	−1.07	0.0384	0.0427	0.752	0.01704	−1.659	0.003	0.0043

^1^ Ratios from EPM: open time/total time; open arms entries/total entries. ^2^ All protein markers were detected in frontal cortex, except for nitrites, which were determined in plasma.

**Figure A1:**
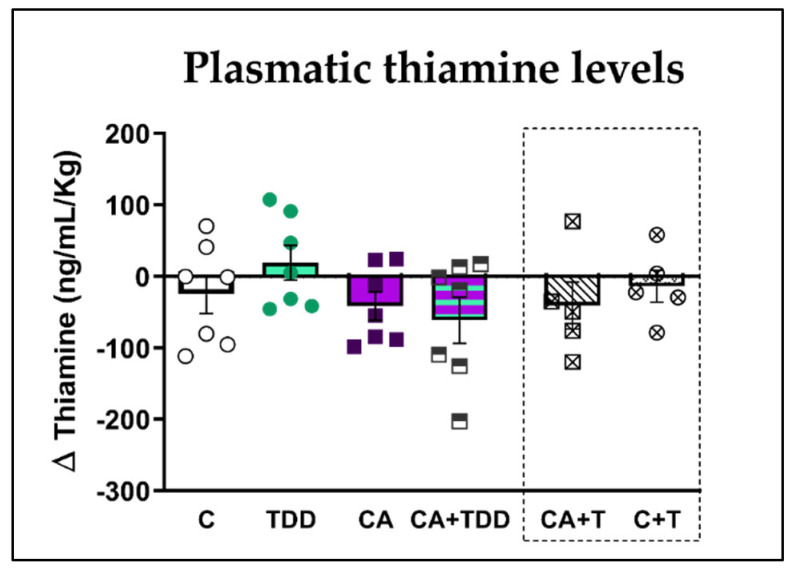
Decrement in plasma levels of total thiamine at the end of the protocol (36 weeks) versus intermediate status (week 12) in adult rats. Results normalized by body weight (ng/mL data divided by kilogram). Data are expressed as mean ± S.E.M.

**Figure A2:**
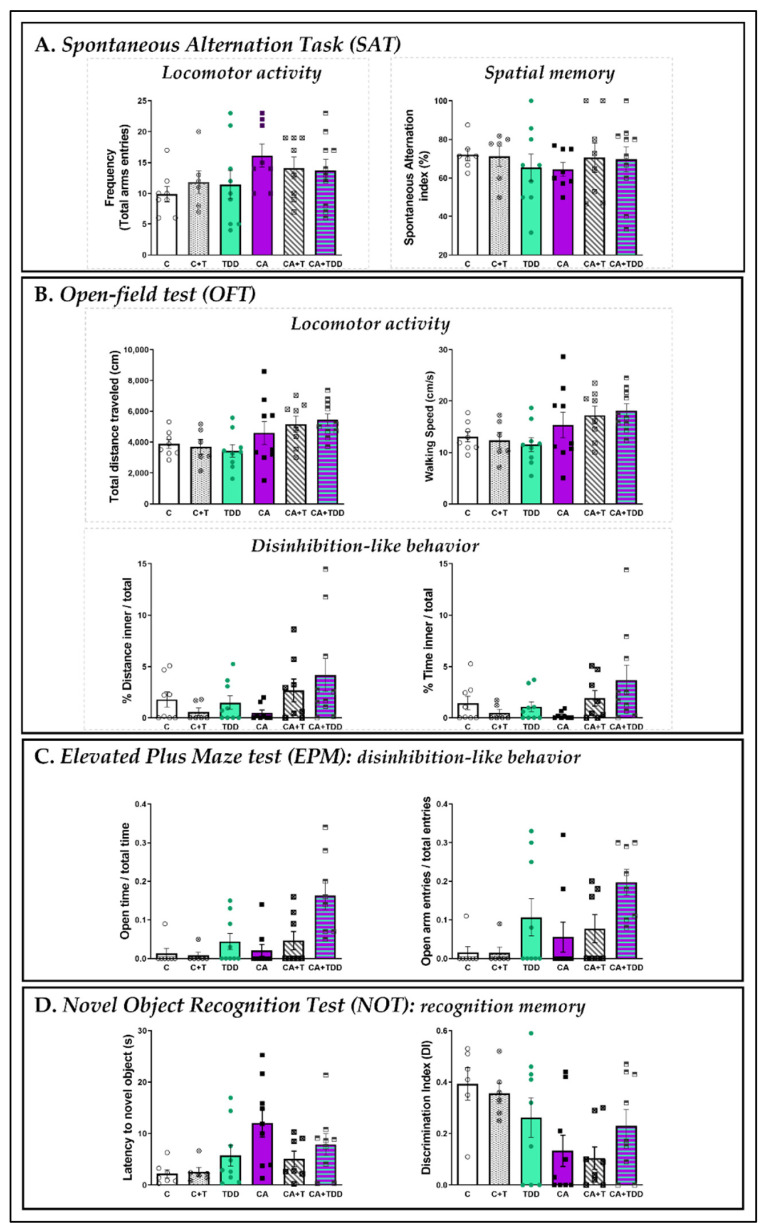
Behavioral data including the oral thiamine-supplemented groups (C+T, CA+T). (**A**) Spontaneous alternation task (SAT). (**B**) Open field (OFT) testing locomotion and disinhibition. (**C**) Elevated plus maze (EPM). (**D**) Effect on memory abilities tested by NOT. Mean ± SEM (*n* = 6–10). Data analyzed by unpaired Student’s t-test or Mann–Whitney test to compare CA+T vs. CA group (*p* > 0.05, n.s.).

**Figure A3:**
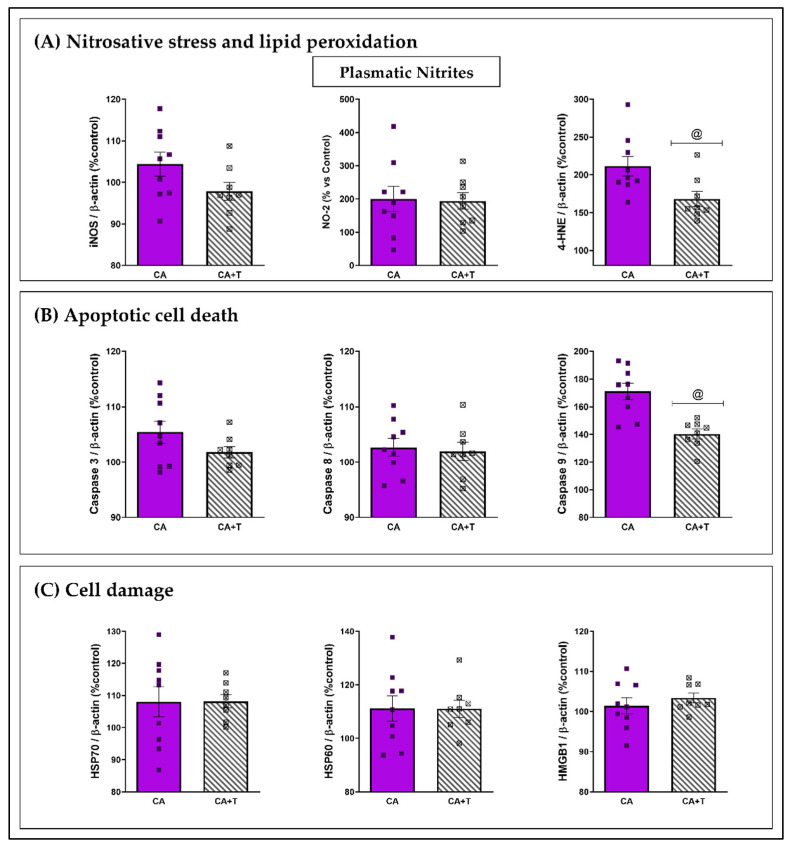
Biochemical data from the oral thiamine-supplemented CA group. Graphs indicate protein levels of markers in the frontal cortex and plasmatic nitrites. Western blot data of the respective proteins of interest were normalized by β-actin and expressed as a percentage of change versus the control group. (**A**) Nitrosative stress markers. Lipid peroxidation was measured by 4-Hydroxynonenal (4-HNE) accumulation. (**B**) Markers of apoptotic cell death: caspase 3, 8 and 9. (**C**) Damage-associated molecular patterns (DAMPS): HSP70, HSP60 and HMGB1. Mean ± SEM (*n* = 8–9). Unpaired Student’s *t*-test or Mann-Whitney were different from CA group at *p* < 0.05.

## Data Availability

The data presented in this study are available on request from the corresponding author.

## Appendix A. Supplemental Discussion of the Physiological Results: Body Weight, Alcohol Intake and Neurological State

In the present work with young growing rats, we observed that the body weight of the CA rats was always significantly lower than that in the controls. In this regard, prolonged alcohol consumption is related to an increased risk of nutritional deficiency or malnutrition. Alcohol’s effects on the stomach and intestines may decrease the absorption and utilization of nutrients while increasing its excretion. In accordance with our findings, it has been shown that prolonged alcohol intake may result in lower body mass gain in animal experiments in comparison with animals that do not receive alcohol (reviewed in [57]). The reduced body weight gain observed in young rats drinking alcohol could be due to loss of appetite with lower dietary intake or increased energy expenditures [58]. Simultaneously, in the case of growing organisms, the influence of alcohol consumption on the growth hormone level may be especially important and may be decreased by ethanol consumption, with a resultant reduction in bodily growth (reviewed in [57]). In addition, its known that TD reduces food consumption and body weight in rodents [18,59], which is in accordance with our results. Regarding this, it has been proposed that thiamine, as well as alcohol, could play a main role in regulating the increase in energy expenditure [59].

Regarding alcohol intake, early ethanol exposure leads to a greater propensity for the development of AUDs [60]. In the present work, the age of the animals appeared to influence the observed pattern of alcohol consumption. Adolescent onset (around PD 40) of chronic drinking led to higher alcohol intake compared with the later stages. This is consistent with previous studies on alcohol exposure in rats, where adolescents drank more and displayed an increased metabolic rate compared with adult rats [23].

In relation to neurological signs, the characteristic symptomatology induced by TD has been extensively demonstrated in rodents, displaying ataxia (i.e., gait problems), loss of righting reflex, and the emergence of seizures and opisthotonos [61,62,63]. In our previous study with TD-induced WKS models [18], we observed clear motor impairments related with cerebellar dysfunction between 14 and 16 days of TD, but at 12 days, the animals were still in a pre-symptomatic state. In accordance with this work, the 12 days of TD diet appeared to not be enough to cause severe neurological signs, even in combination with CA treatment. This could be associated with the absence of alterations in the cerebellum of these animals (unpublished results). Although there is previous evidence pointing to the cerebellum as a vulnerable structure to alcohol and TD [64,65], here, we did not find the TLR4-neuroinflammatory signature in any of the treatment conditions. Future studies will be carried out in this regard.

Nevertheless, we also consider it interesting to point out an impaired sensitivity and reactivity to sound stimuli found in some alcohol-treated animals. Accordingly, the intake of alcohol has been related to a decrease in response to a variety of external stimulus [66]. We found a slight deficit in the startle reflex in animals exposed to alcohol, which were slower to react (i.e., spent more time to manifest the startle reflex than the controls after the noise (clap or finger snaps), needing repetition of the stimulus on several occasions to achieve a response). In line with our results, previous studies reported that ethanol markedly affects the startle response in rats by a possible mechanism involving central dopaminergic and serotoninergic neurons [66].

## Appendix B. Thiamine: Plasma Levels and Oral Supplementation Treatment. Results and Conclusions

Analysis of the levels of thiamine in the plasma from the initial (1 month) and intermediate (3 months) time points showed no significant differences between nor within the controls or CA groups (week 4: control = 55.5 ± 6.28 ng/mL; CA = 55.82 ± 7.31 ng/mL versus week 12: control = 49.37 ± 4.8 ng/mL; CA = 52.06 ± 9.55 ng/mL) (two-way RM ANOVA, interaction: F _(1, 20)_ = 0.03686; effect of time: F _(1, 20)_ = 0.6462; effect of treatment: F _(1, 20)_ = 0.03426; *p* > 0.05, n.s).

However, in the final measurement, after a long period (9 months) of exposure to alcohol and after TD diet treatment, we found a trend toward a decrease in the total thiamine levels due to an alcohol-induced effect. In Figure A1, the thiamine increment (Δ Thiamine) was calculated for each group between the two time points (final and intermediate levels) during the adult stage (because of similar metabolism). Due to significant weight loss in the TDD and CA+TDD animals, the values of the thiamine levels were weight-corrected (ng/mL per kilogram) (two-way ANOVA, main effect of alcohol F _(1, 24)_ = 3.411, *p* = 0.0771).

As it has been previously mentioned, chronic alcohol consumption leads to nutritional deficiency in the form of TD. Our results showed a trend toward a decline in the thiamine levels in the plasma of CA rats. Nevertheless, we analyzed the total levels of thiamine and not its active form (thiamine diphosphate (TDP)), as has been studied in other works [44,52]. Zahr et al. (2014) [67] reported that the total thiamine concentrations were not sensitive to thiamine deprivation, but they found significant lower levels of its metabolite TDP. Accordingly, it would be expected that the active forms of this vitamin would be more affected by the treatments.

In the present experiment, in addition to the four experimental groups reported in the Results section, we also included a group receiving CA with oral thiamine supplementation to deduce whether the effects of long-term alcohol exposure could be attenuated with prophylactic treatment.

The experiment was performed with all six groups simultaneously, as shown in Figure A1 and Figure A2. However, to make it easier for the reader to visualize the results, we simplified them by comparing only the CA+T versus CA group, as represented for the rest of the data in Figure A3. There was no significant general improvement due to oral thiamine treatment in either the behavioral or biochemical assessments (Figure A2 and Figure A3). The 4-HNE and caspase-9 levels decreased in the CA+T group compared with the CA group (Figure A3A–C, respectively), but we are very cautious when considering the effectiveness of the oral thiamine supplementation treatment in light of the overall data.

Here, we also include the physiological data. Regarding the body weight throughout the 36 weeks of experimentation for the C+T and CA+T groups, these animals were gaining weight in a similar way to the control and CA groups, respectively. During the first interval (weeks 1–12), the mean weight of the C+T group was 361.4 ± 3.1 g, and for the CA+T group, it was 327.8 ± 16.5 g. Finally, the C+T and CA animals reached a weight of 525.3 ± 12.3 g and 467.3 ± 26 g, respectively.

Supplementing thiamine apparently did not restore the total thiamine levels in the CA+T-treated rats (Figure A1).

In relation to CA+T alcohol intake, these animals displayed a consumption pattern similar to the CA group, with an initial consumption higher (weeks 1–12: 10.8 ± 0.6 g/Kg) than in the final stage (7.5 ± 0.4 g/Kg). Likewise, the global BEC mean was of 67.8 ± 8.3 mg/dL.

Thus, this indicates that the oral treatment at this dose of thiamine did not prevent the effects of CA that could be caused by alcohol-induced TD itself. Although there are no universally accepted guidelines, the recommended treatment of WKS consists of thiamine administration, preferably by an intravenous route, followed by oral administration [68]. As described above in the introduction of this article, CA exposure can impair the gastric absorption and cellular utilization of thiamine. However, the oral route is suitable for repeated and prolonged use, as it is the simplest and most convenient as well as pain-free.

In addition to thiamine supplementation, other oral treatment alternatives have been proposed. For example, benfotiamine is pro-vitamin B1 with greater absorption and bioavailability compared with orally administered thiamine, producing higher thiamine (and also thiamine mono and diphosphate) concentrations after alcohol exposure [69].
